# A duplex fluorescent quantitative PCR assay to distinguish the genotype I, II and I/II recombinant strains of African swine fever virus in China

**DOI:** 10.3389/fvets.2024.1422757

**Published:** 2024-06-04

**Authors:** Zhiqiang Hu, Ranran Lai, Xiaogang Tian, Ran Guan, Xiaowen Li

**Affiliations:** ^1^Shandong Engineering Laboratory of Pig and Poultry Healthy Breeding and Disease Diagnosis Technology, Xiajin New Hope Liuhe Agriculture and Animal Husbandry Co., Ltd., Dezhou, China; ^2^College of Animal Science, Xichang University, Xichang, China; ^3^College of Veterinary Medicine, Northwest A&F University, Xianyang, China; ^4^China Agriculture Research System-Yangling Comprehensive Test Station, Yangling Besun Agricultural Industry Group Corporation Co., Ltd., Xianyang, China

**Keywords:** duplex fluorescent quantitative PCR, genotype identification, ASFV genotype I, ASFV genotype II, ASFV genotype I/II recombinant strain

## Abstract

African swine fever (ASF) is a severe, hemorrhagic, and highly contagious disease caused by the African swine fever virus (ASFV) in both domestic pigs and wild boars. In China, ASFV has been present for over six years, with three genotypes of strains prevalent in field conditions: genotype I, genotype II, and genotype I/II recombinant strains. In order to differentiate among these three ASFV genotypes, a duplex fluorescent quantitative PCR method was established using specific probes and primers designed based on viral genes MGF_110-1L and O61R from ASFV strains reported in the GenBank database. Following optimization of reaction conditions, a duplex fluorescent quantitative PCR method was successfully developed. This method demonstrated no cross-reactivity with porcine epidemic diarrhea virus (PEDV), transmissible gastroenteritis virus (TGEV), porcine reproductive and respiratory syndrome virus (PRRSV), classic swine fever virus (CSFV), porcine pseudorabies virus (PRV), porcine circovirus 2 (PCV2), porcine circovirus 3 (PCV3), highlighting its specificity. Sensitivity analysis revealed that the limits of detection (LODs) of this method were 2.95 × 10^−1^ copies/μL for the MGF_110-1L gene and 2.95 × 10^0^ copies/μL for the O61R gene. The inter- and intra-group coefficients of variation were both <1%, indicating high reproducibility. In summary, the establishment of this duplex fluorescent quantitative PCR method not only addresses the identification of the ASFV recombinant strains but also allows for simultaneous identification of the three epidemic genotype strains.

## Introduction

ASF is an acute, hemorrhagic, highly contagious disease caused by ASFV in both domestic pigs and wild boars. Due to its exceptionally high fatality rate, it has been classified as a notifiable animal disease by the World Organisation for Animal Health (WOAH) (1). ASFV was initially identified and isolated in Kenya in 1921, and it was introduced into China in 2018, with subsequent reports of ASF outbreaks in other Asian countries (2, 3). ASFV is a member of the family *Asfarviridae* and is the sole arthropod-borne DNA virus in the genus *Asfivirus*. Classification of ASFV into 24 genotypes is based on the C-terminal sequence of the p72 gene (4). At present, China exhibits a predominance of three primary genotypes including genotype I, genotype II, and genotype I/II recombinant strains. The genotype II strains, first detected in China in 2018, demonstrate significant genetic similarity to the Georgia strain and is characterized by its high virulence, resulting in a clinical fatality rate of up to 100% (3). The EP402R gene, a key virulence factor of genotype II strains, encodes the CD2v protein and is responsible for the hemadsorption (HAD)-positive phenotype (3, 5). In contrast, genotype I strains, characterized by a low virulence phenotype of ASFV lacking the HAD phenotype, were identified in field samples from China in 2021 (6). Genotype I strains primarily manifest as chronic clinical symptoms without causing pig mortality, which is attributed to the absence of the MGF_505/360 genes in their genome and impaired expression of the EP402R gene (6, 7). Genotype I/II recombinant strains were first reported in 2023, demonstrating stronger virulence and higher transmissibility compared to genotype I and II strains (8). The recombinant strains are with mosaic genomes composed of 56.5% Georgia07-like genotype II virus and 43.5% NH/P68-like genotype I virus (8). Notably, the genotype-determining gene B646L of the recombinant strain is from genotype I virus, whereas the EP402R gene encoding CD2v is from genotype II virus (8). There is a lack of effective vaccines or drugs for combating ASFV, necessitating a focus on early and precise detection of ASFV in clinical samples as the primary method of prevention. The widely utilized approaches for detection and identification of ASFV are RT-qPCR methods. Due to the presence of multiple genotype strains, the accurate identification of genotypes through qPCR facilitates an initial assessment of the virulence of the infecting strains, thereby enabling the development of targeted control strategies against ASF outbreaks. Various methods have been applied for genotyping of ASFV, including qPCR (9), recombinase polymerase amplification (RPA)/recombinase-aid amplification (RAA) (10, 11), loop-mediated isothermal amplification (LAMP) (12), clustered regularly interspaced short palindromic repeats (CRISPR) (13) etc., targeting viral genes such as B646L, EP402R, E183L, I177L, MGF505-7R, MGF505-2R, MGF360-12L, and MGF360-14L (14).

However, the emergence of a genotype I/II recombinant strain in China has highlighted the inadequacy of current methods for typing all three genotypes simultaneously. This study presents a dual-fluorescence quantitative PCR method that can identify genotype I, genotype II, and genotype I/II recombinant strains by analyzing their genetic sequence characteristics. This method can be applied in the development of early warning, control, and recovery strategies in ASF outbreaks.

## Methods and materials

### Primers and probes

**Figure 1:**
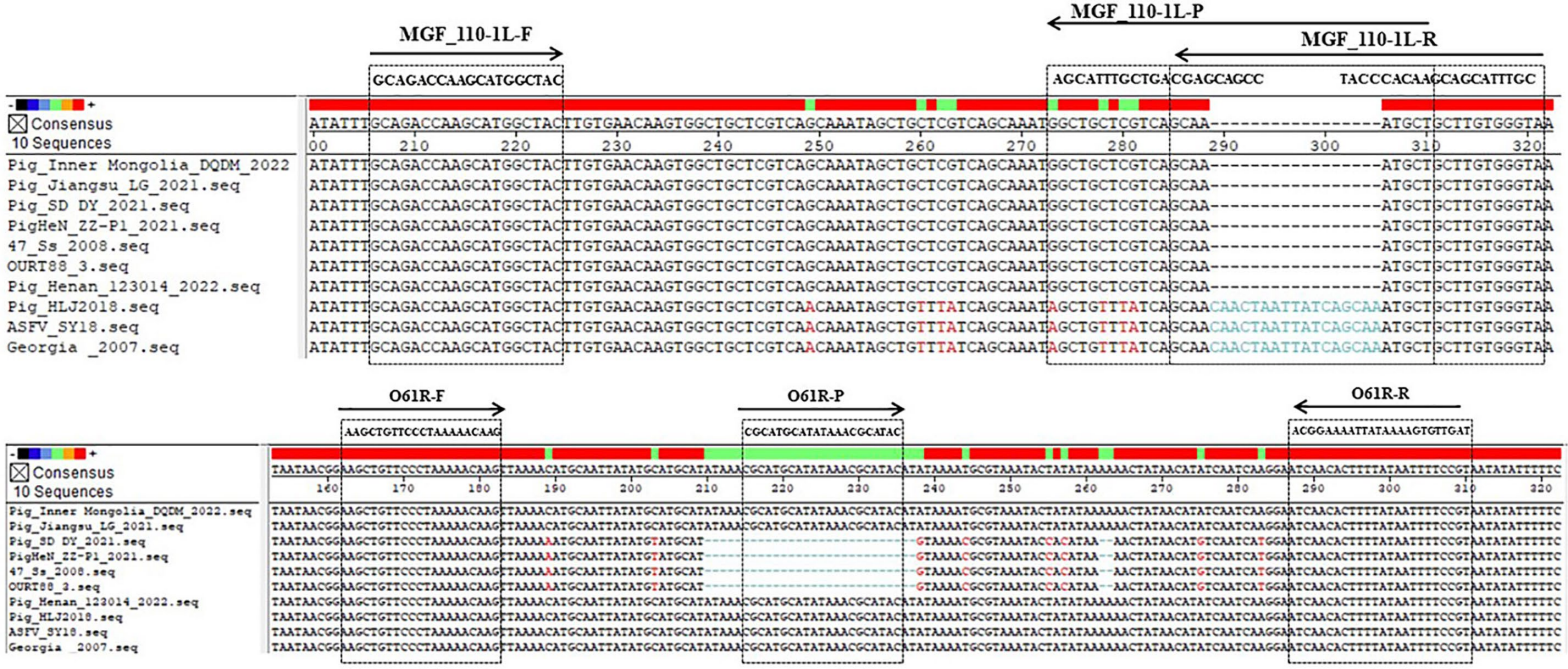
Location of primer and probe sequences in the genomes of different ASFV strains.

Ten ASFV genome sequences were chosen from the NCBI GenBank database, comprising 3 genotype I/II recombinant strains, 4 genotype I strains, and 3 genotype II strains (Table 1). The conservation and variation of sequences between the genotype I/II recombinant strains and other prevalent strains were analyzed using DNAstar Megalign software, as depicted in Figure 1. Primers and probes were designed separately for the genotype I/II recombinant strain with genotype I and genotype II strains, based on conserved regions, utilizing Primer Premier 6 software (Figure 1 and Table 2). Primers of MGF_110-1L-F and MGF_110-1L-R were specifically designed to target conserved regions of ASFV genotype I strains and genotype I/II recombinant strains, with the probe of MGF_110-1L-P emitting FAM fluorescence. Similarly, primers of O61R-F and O61R-R were designed to amplify conserved regions of ASFV genotype II strains and genotype I/II recombinant strains, with the probe of O61R-P emitting VIC fluorescence. These primers and probes were synthesized by Sangon Biotech (Shanghai) Co., Ltd., diluted to a concentration of 10 μM with ddH_2_O, and stored at −20°C.

**Table 1:** Information of the reference strains.

Number	Strains	GenBank number	Year	Genotype
1	Pig_Henan_123014_2022	OQ504954	2022	I/II recombinantion
2	Pig_Inner Mongolia_DQDM_2022	OQ504955	2022	I/II recombinantion
3	Pig_Jiangsu_LG_2021	OQ504956	2021	I/II recombinantion
4	Pig_SD DY_2021	MZ945537	2021	I
5	PigHeN_ZZ-P1_2021	MZ945536	2021	I
6	OURT88_3	AM712240	1988	I
7	47_Ss_2008	KX354450	2008	I
8	ASFV_SY18	MH766894	2018	II
9	Pig_HLJ2018	MK333180	2018	II
10	Georgia_2007	FR682468	2007	II

**Table 2:** Sequences of primers and TaqMan probes.

Name	Sequence (5′–3′)	Fluorescence signal	Product size (bp)
MGF_110-1L-F	GCAGACCAAGCATGGCTAC	—	138
MGF_110-1L-R	TACCCACAAGCAGCATTTGC	—	
MGF_110-1L-P	FAM-AGCATTTGCTGACGAGCAGCC-BHQ1	5′FAM-3′BHQ1	
O61R-F	AAGCTGTTCCCTAAAAACAAG	—	149
O61R-R	ACGGAAAATTATAAAAGTGTTGAT	—	
O61R-P	VIC-CGCATGCATATAAACGCATAC-BHQ1	5′VIC-3′BHQ1	

### Standard plasmid

The pUC57-ASFV standard plasmid was generated through the synthesis and cloning of gene sequences amplified from the MGF_110-1L gene of ASFV genotype I isolate (MZ945537) and the O61R gene of genotype II isolate (MK333180) in GenBank into the pUC57 vector. Quantification of the standard plasmid was performed using a UV–visible spectrophotometer, and copy numbers were determined using a specific formula (15). Subsequently, a 10-fold serial dilution was conducted, resulting in concentrations ranging from 2.95 × 10^9^ to 2.95 × 10^−1^ copies/μL, which were then stored at −20°C for further use.

### Optimization of reaction conditions

Concentrations of primers and probes were optimized by a matrix method. Various concentrations of primers (10 μM) ranging from 0.2 to 0.8 μL each, and probes (10 μM) ranging from 0.1 to 0.4 μL each, along with annealing temperatures between 55°C and 61°C, were tested to achieve the desired optimization. The objective was to minimize the Cq value and maximize the fluorescence intensity (RFU) in the reaction.

### Evaluation of sensitivity and construction of standard curves

Standard plasmids of pUC57-ASFV were utilized as templates for the duplex fluorescent quantitative PCR method, with 10-fold serial dilutions ranging from 2.95 × 10^9^–2.95 × 10^−1^ copies/μL. This method was employed for dual-fluorescence quantitative PCR amplification to generate an amplification kinetics curve and assess the sensitivity. The LODs of the MGF_110-1L gene and the O61R gene were evaluated by plotting the concentration of standard plasmids on the *x*-axis and the cycle threshold (Cq value) on the *y*-axis.

### Evaluation of specificity

The duplex fluorescent quantitative PCR method was employed to utilize cDNA of PEDV, TGEV, PRRSV, CSFV, and DNA of PRV, PCV2, and PCV3 as templates. The pUC57-ASFV standard plasmid served as the positive control, while ddH_2_O was utilized as the negative control to assess the specificity of this method.

### Evaluation of reproducibility

Using standard plasmids of pUC57-ASFV with concentrations ranging from 2.95 × 10^5^ to 2.95 × 10^1^ copies/μL as templates, three batches of repeated tests were performed, with three replicates at each dilution within each batch. Cq values were statistically analyzed to calculate the intra- and inter-group coefficients of variation.

### Clinical sample testing

A total of 96 clinical samples were collected by farmers from pig farms in Shandong and Hebei Province and sent to our lab for testing, comprising 32 serum samples, 51 throat swabs, and 13 environmental wipe samples. DNA extraction was performed on 300 μL of serum, throat swab eluent, or environmental wipe eluent using the NPA-96E Automatic nucleic acid extractors from Bioer Technology Co., Ltd. (Hangzhou, China). Subsequently, 5 μL of the extracted DNA underwent qPCR detection using both the developed duplex fluorescent quantitative PCR and the method recommended by WOAH. The pUC57-ASFV standard plasmid served as the positive control, while ddH_2_O was utilized as the negative control. A Cq value of <40 was considered as a positive result.

## Results

### Optimization of reaction conditions

Reaction conditions were optimized by the matrix method. The optimized 20 μL reaction system was as follows: 10 μL Probe Mix, 0.2 μL each of upstream and downstream primers (10 μM), 0.1 μL probes (10 μM), 4 μL template, and ddH_2_O added to a final volume of 20 μL. The reaction program was as follows: 37°C for 2 min; 95°C for 5 min; 95°C for 10 s, 60°C for 30 s, for 40 cycles.

### Evaluation of sensitivity and construction of standard curves

**Figure 2:**
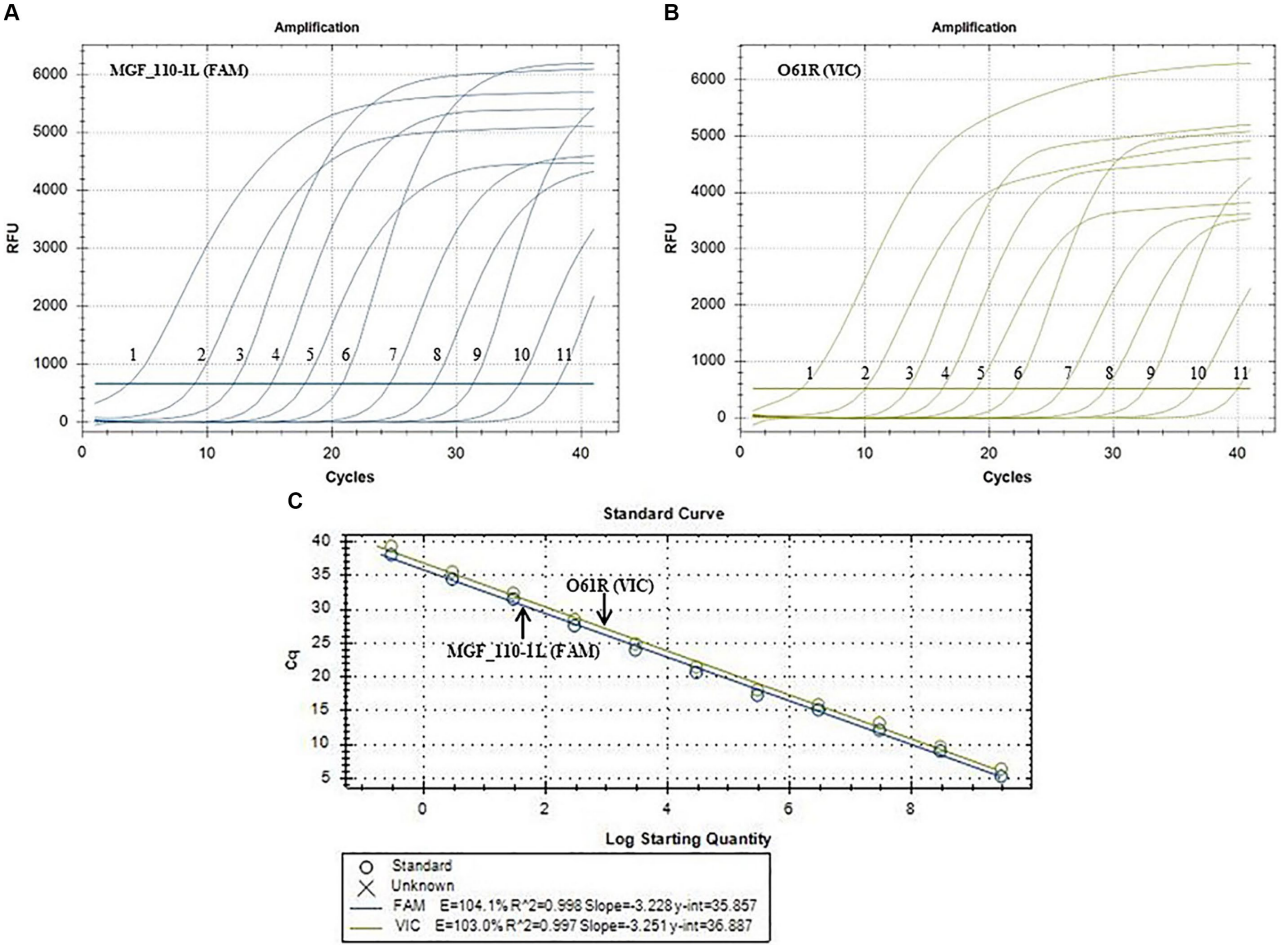
Sensitivity amplification curves and standard curves of the duplex fluorescent quantitative PCR. **(A,B)** The sensitivity amplification curves of MGF_110-1L gene with FAM channel **(A)** and O61R gene with VIC channel **(B)**. Number 1–11: 2.95 × 10^9^–2.95 × 10^−1^ copies/uL. **(C)** Standard curves of both MGF_110-1L gene and O61R gene.

Positive plasmids were utilized as templates for fluorescence quantitative PCR amplification following a 10-fold gradient dilution, resulting in a concentration range of 2.95 × 10^9^–2.95 × 10^−1^ copies/μL. As shown in Figure 2, the LOD for the MGF_110-1L gene was 2.95 × 10^−1^ copies/μL (Figure 2A) and the O61R gene was 2.95 × 10^0^ copies/μL (Figure 2B), demonstrating the excellent sensitivity of the detection method established in this study. Furthermore, standard curves were automatically generated by the fluorescence quantitative PCR instrument. The standard curve for the MGF_110-1L gene exhibited a linear equation of *Y* = −3.228X + 35.857, with a coefficient of determination (*R*^2^) of 0.998 and an efficiency (Eff%) of 104.1%. Similarly, the standard curve for the O61R gene showed a linear equation of *Y* = −3.251X + 36.887, with an *R*^2^ of 0.997 and an Eff% of 103.0%, as illustrated in Figure 2C. The aforementioned results demonstrated a strong linear correlation between the quantity of template and Cq value across the range of diluted concentrations.

### Evaluation of specificity

**Figure 3:**
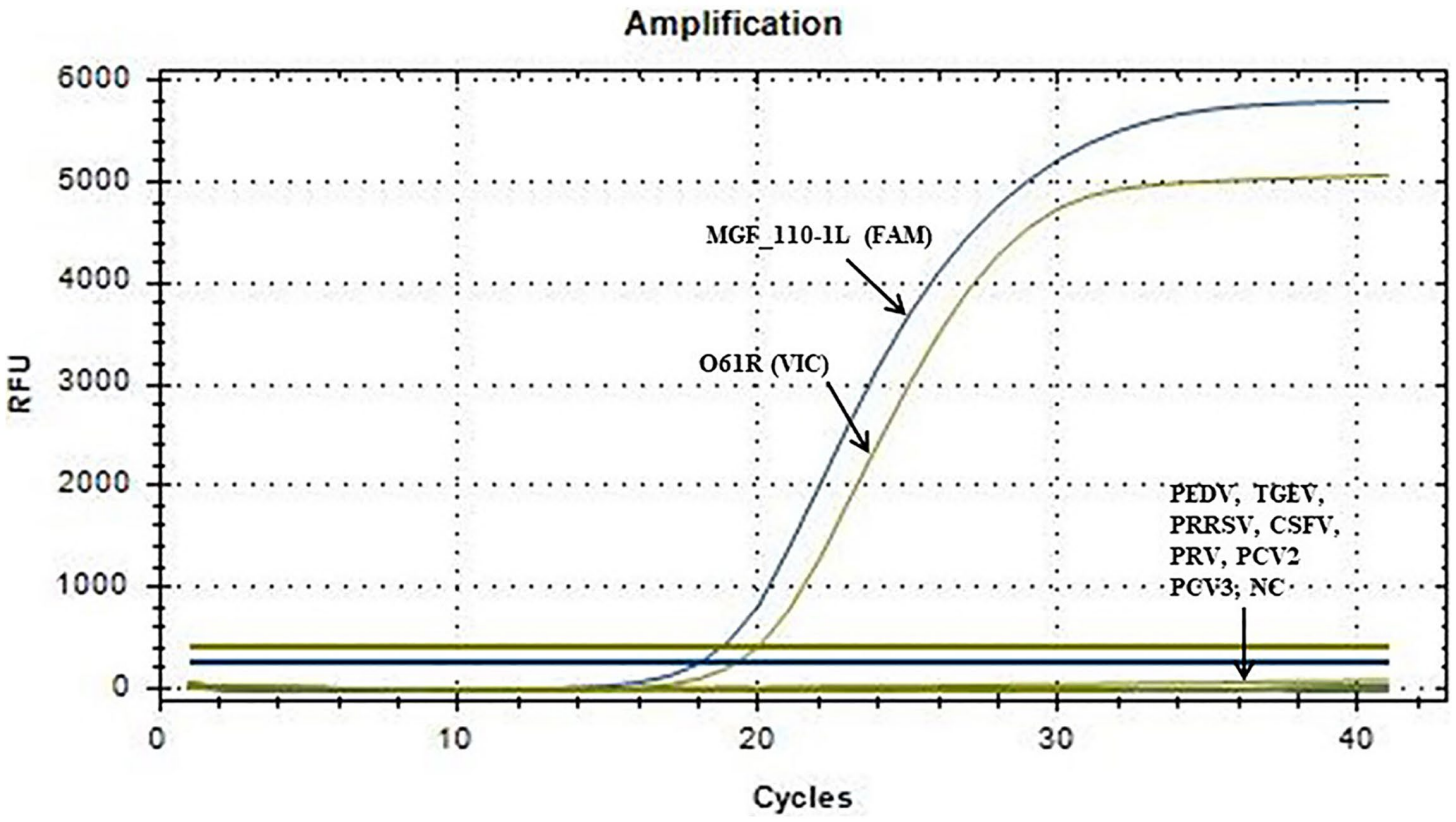
Specific amplification curve of the duplex fluorescent quantitative PCR. No detection signal was obtained for PEDV, TGEV, PRRSV, CSFV, PRV, PCV2, PCV3 or NC.

The optimized reaction protocol was utilized for the detection of nucleic acids from various porcine viruses, including PEDV, TGEV, PRRSV, CSFV, PRV, PCV2 and PCV3. As shown in Figure 3, the result illustrated the absence of amplification curves for the aforementioned pathogens or the negative control, suggesting no cross-reactivity with common porcine viruses.

### Evaluation of reproducibility

As shown in Table 3, the intra-group coefficients of variation ranged from 0.23 to 0.51%, and the inter-group coefficients of variation ranged from 0.12 to 0.4%. The results indicated the excellent reproducibility of this method.

**Table 3:** Intra-reproducibility and intra-repeatability of the MGF_110-1L gene and O61R gene by the duplex fluorescent quantitative PCR.

Target gene	Template concentration (copies /μL)	Intra-assay variation			Intra-assay variation		
		Average value	Standard deviation	CV	Average value	Standard deviation	CV
MGF_110-1L	2.95 × 10^5^	17.73	0.09	0.51%	17.91	0.07	0.39%
	2.95 × 10^4^	20.74	0.05	0.24%	21.49	0.08	0.37%
	2.95 × 10^3^	24.72	0.08	0.32%	24.63	0.05	0.20%
	2.95 × 10^2^	28.06	0.13	0.46%	28.29	0.11	0.39%
	2.95 × 10^1^	31.13	0.13	0.42%	31.66	0.09	0.28%
O61R	2.95 × 10^5^	18.72	0.07	0.37%	18.66	0.06	0.32%
	2.95 × 10^4^	21.89	0.09	0.41%	22.36	0.03	0.13%
	2.95 × 10^3^	25.89	0.06	0.23%	25.63	0.03	0.12%
	2.95 × 10^2^	29.21	0.08	0.27%	29.35	0.09	0.31%
	2.95 × 10^1^	32.30	0.11	0.34%	32.82	0.13	0.40%

### Clinical sample testing

**Figure 4:**
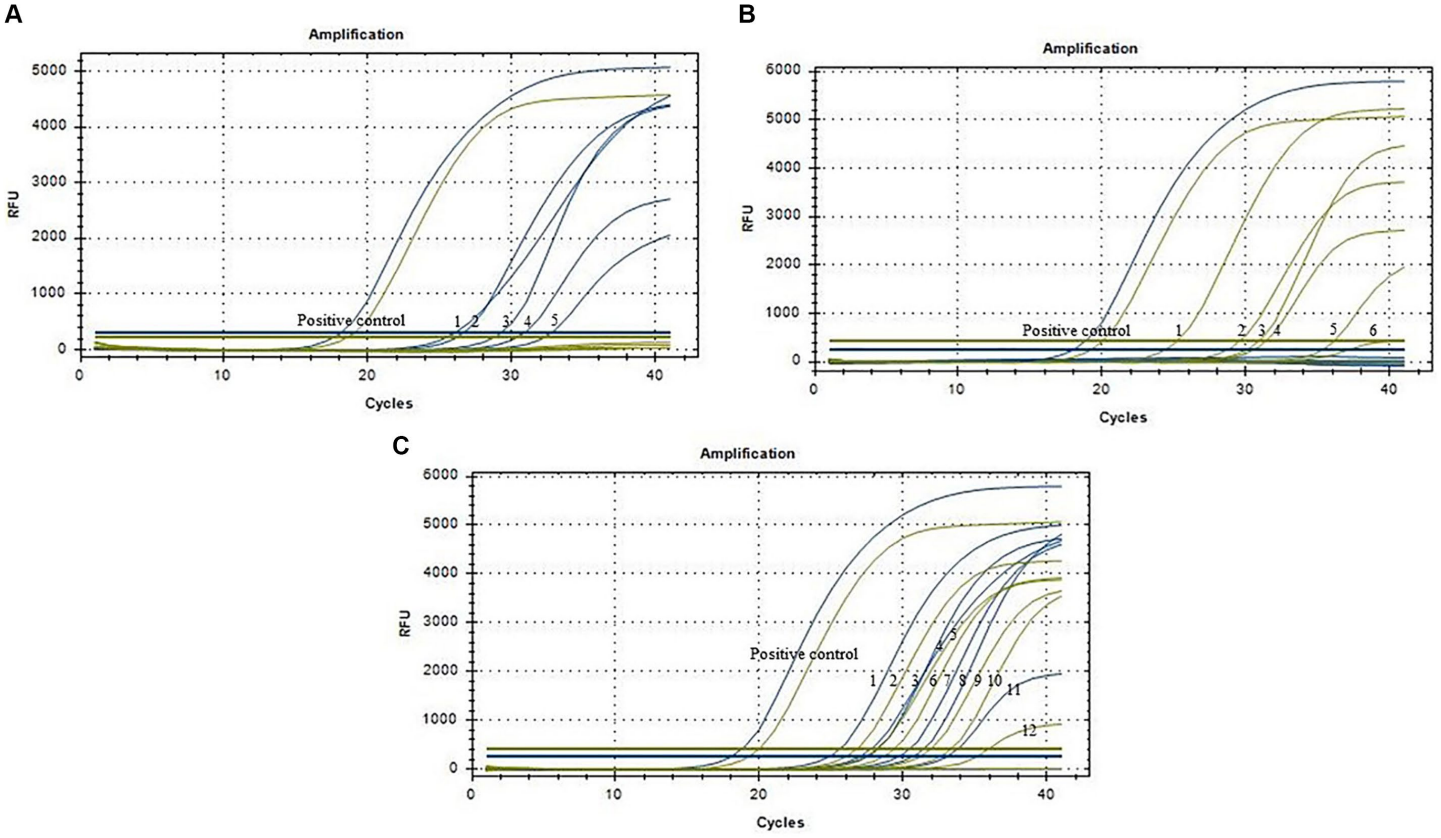
Amplification curves of clinical samples (*N* = 96). **(A)** Five positive samples of ASFV genotype I strains (No. 1–5). **(B)** Six positive samples of ASFV genotype II strains (No. 1–6). **(C)** Six positive samples of ASFV genotype I and II recombinant strains (No. 1–12). Blue lines represent the MGF_110-1L gene, and yellow lines represent the O61R gene.

Results depicted in Figure 4 indicated that out of the 96 clinical samples, 17 tested positive for ASFV, which was consist with results by the WOAH method. Among these positive samples, 5 were identified as ASFV genotype I strains, 6 as ASFV genotype II strains, and 6 as ASFV genotype I/II recombinant strains. These findings suggested that the method developed in this study can effectively be utilized for the laboratory diagnosis and identification of ASFV genotypes.

## Discussion

ASFV, a DNA virus, demonstrates notable levels of variability and frequency of variant strains. In China, three genotypes of ASFV have been identified: genotype I, genotype II, and genotype I/II recombinant strains (3, 6, 8). Additionally, the emergence of commercial gene-deleted vaccines, such as the ASFV-△I177L vaccine, has raised safety concerns (16–18) to the pig industry. Consequently, the genetic diversity of these viral strains has spurred researchers to continually enhance and innovate detection methods and technologies.

There have been some methods currently in use for identification of ASFV genotypes (14). Among these methods, qPCR is commonly favored by large-scale farms due to its high sensitivity and relatively low cost. Cao et al. (19) have devised a qPCR assay that specifically targets the B646L gene for distinguishing between ASFV genotype I and genotype II strains, achieving a LOD of 10 copies per reaction. It was observed that there was only a single base variation in PCR products between ASFV genotype I and genotype II strains in this method. However, recombinant strains are categorized as genotype I based on their B646L gene sequence (8), indicating that the B646L gene may not be a reliable target for distinguishing among the three ASFV genotypes. Additionally, Gao et al. (20) have developed a dual real-time PCR assay to differentiate between genotype I and genotype II by targeting two genes, B646L and E183L, with LODs of 1.07 × 10^2^ copies/μL for B646L and 3.13 × 10^4^ copies/μL for E183L. It was observed that there was only a single base disparity in the E183L gene in PCR products and no disparity in the B646L gene in PCR products between genotype I and genotype II strains, potentially constraining the sensitivity of this methodology. To concurrently identify the three genotypes and enhance sensitivity, a comparative analysis was performed in this study on the sequences of genotype I, genotype II, and genotype I/II recombinant strains to elucidate the variations and similarities among them. Subsequently, the MGF_110-1L gene and the O61R gene were selected for additional scrutiny. There was a difference of 9 bases in the MGF_110-1L gene PCR products between genotype I or I/II recombinant strains and genotype II strains, and a difference of 21 bases in the O61R gene PCR products between genotype II or I/II recombinant strains and genotype I strains (Figure 1). The LODs for this method were 2.95 × 10^−1^ copies/μL for the MGF_110-1L gene and 2.95 × 10^0^ copies/μL for the O61R gene, which were lower than previously reported values (19, 20). Additionally, this method represents the latest approach capable of distinguishing among genotype I, genotype II, and genotype I/II recombinant strains concurrently. Furthermore, the target genes offered have potential applications in clinical detection for large-scale farms and could also contribute to the development of on-site detection methods when integrated with complementary techniques like LAMP, CRISPR and so on.

The methodology outlined in this study is primarily suited for two key applications. Firstly, it enables precise identification of positive samples obtained from pigs exhibiting suspected clinical symptoms, including throat swabs, blood, and tissue samples, facilitating accurate virus assessment by farmers and veterinarians in the early stages of infection. Additionally, ASFV can survive in the environment for extended periods and can be transmitted through contaminated materials (1, 21). Therefore, precise identification of samples pertaining to the surroundings of swine farms can aid farmers and veterinarians in implementing specific biosecurity measures prior to the onset of infection on the farm, thereby averting the occurrence of ASFV.

In conclusion, the establishment of this duplex fluorescent quantitative PCR method not only addresses the deficiency in identifying recombinant strains but also allows for the simultaneous identification of the three genotypes. This provides a theoretical basis for the formulation of targeted prevention and control strategies against ASFV outbreaks.

## Data availability statement

The raw data supporting the conclusions of this article will be made available by the authors, without undue reservation.

## Ethics statement

This study uses samples obtained from commercial pig farms. Dezhou Animal Disease Prevention and Control Center did not require the study to be reviewed or approved by an ethics committee because the clinical samples used were provided by farmers from pig farms for ASFV diagnostic detection. Written informed consent was obtained from the owners of the animals to use the clinical samples.

## Author contributions

ZH: Visualization, Formal analysis, Writing – review & editing, Writing – original draft, Conceptualization. RL: Resources, Methodology, Data curation, Writing – review & editing, Writing – original draft. XT: Writing – review & editing, Software, Resources, Methodology, Data curation. RG: Writing – review & editing, Software, Resources, Methodology, Data curation. XL: Writing – review & editing, Writing – original draft, Supervision, Funding acquisition, Conceptualization.
